# Effect of Moderate Aerobic Exercise on Complement Activation Pathways in Polycystic Ovary Syndrome Women

**DOI:** 10.3389/fendo.2021.740703

**Published:** 2022-02-17

**Authors:** Manjunath Ramanjaneya, Ibrahem Abdalhakam, Ilham Bettahi, Milin Bensila, Jayakumar Jerobin, Myint Myint Aye, Meis Alkasem, Thozhukat Sathyapalan, Stephen Lawrence Atkin, Abdul-Badi Abou-Samra

**Affiliations:** ^1^ Qatar Metabolic Institute, Interim Translational Research Institute, Academic Health System, Hamad Medical Corporation, Doha, Qatar; ^2^ Department of Academic Endocrinology, Diabetes and Metabolism, Hull York Medical School, Hull, United Kingdom; ^3^ Post Graduate Studies and Research, Royal College of Surgeons in Ireland Bahrain, Adliya, Bahrain

**Keywords:** PCOS, T2D, complement-related proteins, *M* value, VO_2max_, exercise

## Abstract

**Background:**

The complement system is pivotal in host defense mechanisms, protecting against pathogenic infection by regulating inflammation and cell immunity. Complement-related protein activation occurs through three distinct pathways: classical, alternative, and lectin-dependent pathways, which are regulated by cascades of multiple proteins. Complement activation is recognized in polycystic ovary syndrome (PCOS) to be associated with obesity and insulin sensitivity. Exercise reduces insulin resistance and may help reduce obesity, and therefore, this study was undertaken to determine the effect of exercise on the activation of complement-related proteins in PCOS and control women.

**Subjects and Measurements:**

In this study, 10 controls and 11 PCOS subjects who were age- and weight-matched underwent an 8-week supervised exercise program at 60% maximal oxygen consumption. Weight was unchanged though insulin sensitivity was increased in PCOS subjects and controls. Fasting baseline and post-exercise samples were collected and 14 complement-related proteins belonging to classical, alternative, and lectin-dependent pathways were measured.

**Results:**

Baseline levels of complement C4b and complement C3b/iC3b were higher in PCOS (*P* < 0.05) compared with controls. Exercise reduced complement C1q (*P* < 0.05), C3 (*P* < 0.001), C4 (*P* < 0.01), factor B (*P* < 0.01), factor H (*P* < 0.01), and properdin (*P* < 0.05) in controls, but not in PCOS women.

**Conclusion:**

Exercise induced complement changes in controls that were not seen in PCOS subjects, suggesting that these pathways remain dysregulated even in the presence of improved insulin sensitivity and not improved by moderate aerobic exercise.

**Clinical Trial Registration:**

ISRCTN registry, ISRCTN42448814.

## Introduction

Polycystic ovarian syndrome (PCOS) is one of the most common endocrine illnesses affecting 5%–7% of women of reproductive age (1). Insulin resistance, obesity, and dyslipidemia, leading to increased cardiovascular risk and type 2 diabetes (T2D), are common features seen in PCOS women (1–3). Seventy-five percent of women diagnosed with PCOS exhibit obesity, with the majority showing increased central adiposity (4, 5). This PCOS phenotype is associated with hyperandrogenism and insulin resistance, resulting in a higher frequency of impaired glucose tolerance (IGT), T2D, and metabolic syndrome in women with PCOS (4–6). Despite the high prevalence of PCOS, its underlying pathophysiology and etiology still remain unknown, and its management in clinical practice is disjointed between gynecologists, endocrinologists, and general practitioners.

The complement system controls inflammation and comprises three activation pathways: classical, alternative, and lectin, with 50 proteins accounting for around 15% of the globulin fraction (7, 8). Antigen–antibody immune complexes, acute-phase proteins, and apoptotic and necrotic cells like C-reactive protein all activate the classical pathway. The lectin pathway identifies patterns of carbohydrate ligands on the surface of microorganisms by using mannose-binding lectins and ficolin. The alternative pathway is constitutively active in the typical host at low levels in preparation for rapid activation upon stimulus (8). In both the lectin and classical pathways, convertases require the cleavage of C2 and C4 (9) that necessitates the use of factors B and D (9). Both the classical and alternative activation pathways require complement C3. The immune system is impaired by a C3 deficit, making the body more susceptible to infection (10). Traditionally, C1s splits the C4 component in two halves, C4a and C4b. C4b connects to the cell membrane after binding to C2, divided in two subunits, C2a and C2b. Attributable to the serine protease activity of the C2a portion, a heterodimer known as classical C3 convertase is generated by merging the two C4b and C2a elements. As one of the steps of the healthy process, C3 convertase is needed to separate C3 protein further into C3a and C3b elements, which also exists in the lectin activation pathway (11, 12). Factor H and properdin are important in the regulation of alternative pathway activation: factor H acts as an inhibitor, dissociating C3 convertase; elevated levels of both indicate alternative pathway dysregulation, whereas properdin acts as a stabilizing agent of C3 convertase, resulting in prolonged complement activation (9). Only a few studies have examined complement-related protein levels in women with PCOS and reported higher factor D (13) and C3a levels (14, 15) and higher (15, 16) or no difference (17, 18) in C3 levels compared with matched controls. However, basal complement C3 levels are increased in insulin resistance with increased factor H levels associated with obesity in PCOS (9).

Physical activity is a natural stimulus that affects defensive and immune systems in both primary humoral and cellular immunity, influencing complement system activation (19). This interaction is complicated since routine moderate-intensity exercise will activate the immune system, while repetitive high-intensity activity (with inadequate recovery) may inhibit the immune system (20–23). Furthermore, strenuous exercise exertion may trigger oxidative stress with the release of heat shock proteins, catecholamines, cortisol, and insulin-like growth factor 1 (IGF-1) (20, 24), all of which may lead to immune stimulation or suppression based on other co-factors. Given that there are no prior reported studies on the effects of exercise on complement-related protein expression, this study was undertaken in weight-matched and age-matched women with and without PCOS before and after aerobic exercise of moderate duration.

## Methodology

### Study Participant Recruitment and Exercise Protocol

The diagnosis of PCOS was established according to the Rotterdam criteria (13). Ten controls and 11 PCOS subjects were recruited as described previously (25). All the study participants attended fasting for baseline measurements. Following this, the study participants were enrolled in a supervised exercise program that consisted of 1-h supervised exercise three times per week for 8 weeks in the Department of Sports, Health and Exercise Science, University of Hull.

Where possible, each session was 1 h in duration depending on their ability to complete the sessions with no complications. The program used either a Woodway ELG55 motorized treadmill (Woodway, Weil am Rhein, Germany) or an HP Cosmos Pulsar Treadmill (H/P/Cosmos) with the same protocol. Participants performed all sessions on a motorized treadmill working at or as closely as possible to 60% VO_2max_. VO_2_/kg was measured (using an Oxycon Pro Metabolic System Jaegger, Hoechberg, Germany) after the warmup, which lasted for 5 min at 4.5 km h^−1^ and for a period of 10 min in order to confirm the appropriate exercise intensity. The intensity of exercise was then adjusted by altering the speed of the treadmill if this value was not within ±2.5% of the target oxygen uptake. Following this 10-min gas collection, the facemask was withdrawn with the speed of the treadmill remaining as it was. A further gas collection was made at 40 min to confirm the desired intensity for a 5-min period. If this intensity was out of range, then the treadmill speed was once again altered if required. Heart rate (HR) and rate of perceived exertion (RPE) (26) were monitored every 15 min throughout the session. If participants felt that they could not continue with the exercise for reasons such as injury or fatigue, they were able to stop at any time if necessary. Likewise, if it meant reducing the intensity for a period of time in order for them to recover, then this was permitted; otherwise, the intensity remained at the level predetermined. Each session ended with a 5-min cool down at 4.5 km h^−1^, and participants would then be free to leave once HR returned to within 120% of basal levels. During each exercise session, heart rate and inspired/expired gas fractions were continuously monitored and heart rate equivalent to 60% of baseline VO_2max_ was achieved during each session of exercise training (27). Insulin sensitivity was measured using the gold standard euglycemic clamp technique as described previously (28, 29). Within a week following the completion of exercise protocol, all participants were invited to provide blood samples and underwent insulin sensitivity test.

### Ethical Approval

The study was approved by the Yorkshire and the Humber Research Ethics Committee (reference number 10/H1313/44) and The Medical Research Center at Hamad Medical Corporation (reference number RP #17180/17). All study participants gave their written informed consent prior to participation in the study.

### Complement-Related Protein Measurements

Plasma levels of human complement-related proteins were measured using MILLIPLEX MAP Kit Human Complement Magnetic Bead Panels 1 and 2 (HCMP1MAG-19K and HCMP2MAG-19K, Merck Millipore, USA) according to the instructions of the kit manufacturer as described previously (30). Protein levels in the plasma samples were quantified using 5PL logistic regression algorithms that are built into the Bio-Plex Manager 6 software, which was used for the quantification of all plasma samples with reference to the standards provided by the kit manufacturer using overnight incubation protocol. The samples were run on a Bio-Plex 200 (Bio-Rad, Hertfordshire, UK) instrument. The plasma samples were diluted 200 times for the measurement of complement panel-1 proteins (C2, C4b, C5, C5a, C9, factor D, mannose-binding lectin, and factor I) and 40,000 times for the measurements of complement panel 2 (C1q, C3, C3b/iC3b, C4, factor B, factor H, and properdin)-related proteins to attain the levels of the proteins within the reference range of the standard curve. The working range and assay precision for different complement-related proteins were reported previously (30).

### Biochemical Measurements

Plasma levels of other variables were measured as reported previously (27). Serum insulin was measured using a competitive chemiluminescent immunoassay (Euro/DPC, Llanberis, UK). Plasma glucose was measured by a Synchon LX 20 analyzer (Bechman-Coulter, High Wycombe, UK). Triglycerides (TG) and total cholesterol measurements were done using Synchon LX 20 analyzer (Beckman-Coulter). The free androgen index (FAI) was calculated by dividing the total testosterone by sex hormone-binding globulin (SHBG) and then multiplying by 100. Serum testosterone (nmol/L) was assessed by high-performance liquid chromatography linked to tandem mass spectrometry (Waters Corporation, Manchester, UK); SHBG (nmol/L) levels were measured by immunometric assay with fluorescence detection on the DPC Immulite 2000 analyzer (Euro/DPC, Llanberis UK). FSH (iU/L) was measured by an Architect analyzer (Abbott Laboratories, Maidenhead, UK); TCH (mmol/L), TG (mmol/L), and HDL (mmol/L) were measured using Synchron LX 20 analyzer (Beckman Coulter); and LDL (mmol/L) was calculated using the Friedewald equation. Plasma glucose was measured using Synchron LX 20 analyzer (Beckman-Coulter). NEFA was measured using an enzymatic colorimetric method (Wako NEFA-H2) on a Konelab20 auto analyzer with a coefficient of variation of 1.4%. All the above measurements were done according to the recommended protocol of the manufacturer and as mentioned previously (25).

### Statistical Analysis

All the data are expressed as mean ± standard deviation (SD). Baseline differences between the control and PCOS and their expression levels after exercise were determined by unpaired Student’s *t*-test. The effects of exercise within and between the control and PCOS groups were determined by general linear model repeated measure ANOVA. This statistical method of analysis compares the exercise intervention effects within groups and the interaction of group and time and also compares between control and PCOS subjects. Spearman bivariate correlation analysis was performed to study the associations between clinical and biochemical measurements and the complement-related proteins. The statistical package SPSS 22.0 software was used for the data analysis and a *P*-value <0.05 (two-tailed) was considered statistically significant.

## Results

### Baseline Measurements

The baseline measurement of both control and PCOS subjects was previously reported (31). The samples used in this study are a subset from a previous study (31) in which weight did not differ between baseline and 8 weeks exercise for both PCOS and control subjects, though insulin sensitivity increased in both groups. In this study, no significant differences in complement C1q, C2, C5, C5a, C3, C4, factor B, factor H, factor D, mannose-binding lectin, properdin, and complement factor I at baseline were shown between PCOS and controls. Only complement C3b/iC3b (*P* = 0.048) and C4b (*P* = 0.036) were found to be significantly higher in PCOS subjects compared with controls (**Table 1**). Data comparing the differences between controls and PCOS groups following exercise showed significant differences for C1q (*P* = 0.005), C3 (*P* = 0.036), C4 (*P* = 0.01), factor B (*P* = 0.004), factor H (*P* = 0.005), properdin (*P* = 0.005), and C4b (*P* = 0.009) (**Table 1**).

**Table 1:** Comparison of baseline and exercise induced changes in complement related proteins in control and PCOS subjects.

Control v PCOS baseline measurements				Control v PCOS post exercise measurements		
*Protein name*	Control	PCOS	Unpaired t-test	Post-exercise control	Post-exercise PCOS	Unpaired t-test
	Mean ± SD	Mean ± SD	P	Mean ± SD	Mean ± SD	P
Complement- C1q (μg/ml)	86.7± 25.8	92.6± 33.3	0.656	57.7 ± 23.1	89.6 ± 21.4	0.005
Complement C3 (μg/ml)	215.9± 127.6	384.2 ± 242.6	0.113	131.1 ± 82.2	266.5 ± 170.3	0.036
Complement C3b/iC3b (μg/ml)	563.4 ± 568.6	1290.7 ± 942.4	0.048	900.6 ± 1287.3	1066.1 ± 741.0	0.736
Complement C4 (μg/ml)	294.7 ± 134.5	400.5 ± 205.8	0.184	211.8 ± 106.7	396.6 ± 175.5	0.011
Complement factor B (μg/ml)	221.9 ± 93.2	237.5 ± 88.0	0.697	151.1 ± 58.0	257.1 ± 84.9	0.004
Complement factor H (μg/ml)	292.6 ±118.9	335.6 ± 113.7	0.408	204.3 ± 76.7	333.9 ± 101.7	0.005
Properdin (μg/ml)	29.8 ± 8.5	34.0 ± 10.7	0.334	21.8 ± 6.7	32.7 ± 8.6	0.005
Complement C2 (μg/ml)	1.2 ± 0.6	1.7 ± 0.8	0.149	1.0 ± 0.5	1.7 ± 1.3	0.176
Complement C4b (μg/ml)	8.4 ± 2.5	10.6 ± 1.6	0.036	8.0 ± 2.0	10.9 ± 2.0	0.009
Complement C5 (μg/ml)	28.7 ± 14.1	25.3 ± 8.9	0.517	21.8 ± 10.6	23.8 ± 10.9	0.668
Complement C5a (μg/ml)	1.0 ± 0.3	1.0 ± 0.8	0.841	1.1 ± 0.8	1.4 ± 1.1	0.716
Complement factor D (μg/ml)	2.4 ± 2.0	2.4 ± 0.8	0.953	2.1 ± 0.8	2.4 ± 0.9	0.829
Mannose-binding lectin (μg/ml)	3.0 ± 2.5	1.8 ± 1.3	0.204	2.7 ± 2.2	2.1 ± 1.5	0.471
Complement factor I (μg/ml)	27.5 ± 9.7	31.7 ± 8.7	0.318	26.3 ± 8.1	31.9 ± 7.9	0.193

Values are represented as means ± SD. P < 0.05 was considered to be statistically significant. Unpaired T-test was performed to compare effects of exercise between groups pre and post exercise.

### Exercise-Induced Changes in Physiological Variables in Control and PCOS Subjects

Following exercise, there were no significant changes in weight and BMI. However, there was a reduction in waist size both within groups (*P* = 0.031) and between groups (*P* = 0.025). Hip size within groups did not differ (*P* = 0.251) but did differ between groups (*P* = 0.045). A trend to an increase in *M*-value was seen within groups (*P* = 0.057) but differed between groups (*P* = 0.016). An increase in VO_2max_ was seen both within groups (*P* = 0.001) and between groups (*P* < 0.0001, **Table 2**).

**Table 2:** Exercise induced changes in physiological variables and complement related proteins within and between control and PCOS subjects.

	Control		PCOS		Within group	Between groups
	Pre- exercise	Post-exercise	Pre- exercise	Post-exercise	P	P
Weight (kg)	69.7±15.5	70.6±16.2	85.4±17.6	85.0±19.6	0.562	0.077
BMI (kg/m2)	25.1±5.2	25.4±5.4	30.8±5.8	30.6±6.3	0.513	0.052
Waist (cm)	78.4±11.9	76.8±13.2	93.5±13.4	91.6±15.7	0.031	0.025
Hip (cm)	97.5±13.0	99.9±13.3	111.6±12.6	111.6±12.8	0.251	0.045
M-Value (mg /Kg/min)	5.0±2.0	5.5±1.9	3.3±0.8	3.9±1.5	0.057	0.016
VO2max (mL/kg/min)	36.3±6.4	39.2±5.8	26.3±4.6	28.7±5.4	0.001	0.000
Complement- C1q (μg/ml)	86.7± 25.8	57.7 ± 23.1	92.6± 33.3	89.6 ± 21.4	0.026	0.059
Complement C3 (μg/ml)	215.9± 127.6	131.1 ± 82.2	384.2 ± 242.6	266.5 ± 170.3	0.019	0.026
Complement C3b/iC3b (μg/ml)	563.4 ± 568.6	900.6 ± 1287.3	1290.7 ± 942.4	1066.1 ± 741.0	0.672	0.343
Complement C4 (μg/ml)	294.7 ± 134.5	211.8 ± 106.7	400.5 ± 205.8	396.6 ± 175.5	0.070	0.029
Complement factor B (μg/ml)	221.9 ± 93.2	151.1 ± 58.0	237.5 ± 88.0	257.1 ± 84.9	0.103	0.043
Complement factor H (μg/ml)	292.6 ±118.9	204.3 ± 76.7	335.6 ± 113.7	333.9 ± 101.7	0.035	0.042
Properdin (μg/ml)	29.8 ± 8.5	21.8 ± 6.7	34.0 ± 10.7	32.7 ± 8.6	0.050	0.031
Complement C2 (μg/ml)	1.2 ± 0.6	1.0 ± 0.5	1.7 ± 0.8	1.7 ± 1.3	0.509	0.096
Complement C4b (μg/ml)	8.4 ± 2.5	8.0 ± 2.0	10.6 ± 1.6	10.9 ± 2.0	0.729	0.010
Complement C5 (μg/ml)	28.7 ± 14.1	21.8 ± 10.6	25.3 ± 8.9	23.8 ± 10.9	0.126	0.876
Complement C5a (μg/ml)	1.0 ± 0.3	1.1 ± 0.8	1.0 ± 0.8	1.4 ± 1.1	0.061	0.903
Complement factor D (μg/ml)	2.4 ± 2.0	2.1 ± 0.8	2.4 ± 0.8	2.4 ± 0.9	0.888	0.972
Mannose-binding lectin (μg/ml)	3.0 ± 2.5	2.7 ± 2.2	1.8 ± 1.3	2.1 ± 1.5	0.674	0.351
Complement factor I (μg/ml)	27.5 ± 9.7	26.3 ± 8.1	31.7 ± 8.7	31.9 ± 7.9	0.922	0.186

Values are represented as means ± SD. P < 0.05 was considered to be statistically significant. General linear Model repeated measures ANOVA analysis was performed to compare effects of exercise within group effects, the interaction of group and time and to compare between control and PCOS subjects. Interaction between time points and the group were not significant.

### Exercise-Induced Changes in Complement-Related Proteins

Exercise effects were analyzed by general linear model repeated measures to compare the effects of exercise within groups and between control and PCOS group effects. Our data showed that exercise significantly reduced the levels of complement C1q (*P* = 0.026) within groups, but between groups, there was no change. C3 (*P* = 0.019) differed both within groups and between groups (*P* = 0.026). C4 and complement factor B did not show any changes within groups; however, the control and PCOS group comparison was significantly altered for C4 (*P* = 0.029) and complement factor B (*P* = 0.043), following exercise. Complement factor H was significantly reduced both within groups (*P* = 0.035) and between groups (*P* = 0.042). Properdin differed significantly between control and PCOS (*P* = 0.031); similarly, complement C4b was also significantly reduced (*P* = 0.042) between group analysis of control and PCOS subjects following exercise. We did not observe any differences for complement C2, C3b/iC3b, C5, C5a, factor D, mannose-binding lectin, and complement factor I both within and between groups following exercise (**Table 2**). The interactions between time points and the groups were found to be similar (data not shown).

### Correlation Analysis of Complement-Related Proteins With Covariates Before Exercise

The association of complement-related proteins with baseline clinical, hormonal, and biochemical parameters was examined using the Spearman correlation coefficient (**Tables 3A, B**). In the control group, complement C3 levels correlated positively with waist circumference (*P* = 0.047), WHR (*P* = 0.042), and TG (*P* = 0.049). C4 (*P* = 0.015) and factor H (*P* = 0.026) also showed a positive correlation with waist circumference. Properdin showed positive correlations with waist circumference (*P* = 0.026) and ALT (*P* = 0.027). Similarly, complement C4b showed positive correlations with weight (*P* = 0.033) and BMI (*P* = 0.042) in controls (**Table 3A**). In the PCOS group, C3 showed a positive correlation with SBP (*P* = 0.042), and C4 showed a positive correlation with SBP (*P* = 0.011) and a negative correlation with TC (*P* = 0.003). Factor B did not show any significant correlations with measured clinical and biochemical variables. Factor H showed a positive correlation with SBP (*P* = 0.007) and a negative association with TC (*P* = 0.024). Properdin showed a positive correlation with SBP (*P* = 0.042) and C4b showed a negative correlation with ALT (*P* = 0.029, **Table 3B**).

**Table 3A:** Spearman rank correlation analysis of complement related proteins with anthropometric and hormonal variables before exercise controls.

	Complement C3		Complement C4		Factor-B		Factor-H		Properdin		Complement C4b	
	r	p	r	p	r	p	r	p	r	p	r	p
Age	0.358	0.31	0.224	0.533	0.272	0.445	0.187	0.603	0.103	0.776	0.006	0.986
Height	-0.128	0.725	-0.072	0.841	-0.045	0.907	0.054	0.88	0.109	0.763	0.054	0.88
Weight	0.369	0.293	0.539	0.107	0.442	0.2	0.515	0.127	0.466	0.173	0.673*	0.033
BMI	0.357	0.31	0.539	0.107	0.49	0.149	0.527	0.117	0.43	0.214	0.648*	0.042
Waist	0.638*	0.047	0.736*	0.015	0.613	0.058	0.693*	0.026	0.693*	0.026	0.62	0.055
Hip	0.369	0.293	0.515	0.127	0.406	0.244	0.478	0.161	0.418	0.229	0.527	0.117
WHR	0.648*	0.042	0.612	0.059	0.49	0.149	0.515	0.127	0.575	0.081	0.478	0.161
SBP	0.595	0.069	0.474	0.166	0.607	0.062	0.565	0.088	0.504	0.136	-0.042	0.907
DBP	0.353	0.316	0.432	0.211	0.445	0.197	0.487	0.152	0.365	0.298	0.408	0.241
PG	-0.231	0.519	-0.256	0.475	-0.073	0.84	-0.103	0.775	-0.189	0.6	-0.128	0.724
Insulin	0.069	0.859	0.139	0.722	0.228	0.556	0.228	0.556	0.119	0.761	0.149	0.703
NEFA	0.49	0.149	0.442	0.2	0.369	0.293	0.393	0.259	0.43	0.214	-0.042	0.907
M-Value	-0.43	0.214	-0.393	0.259	-0.49	0.149	-0.527	0.117	-0.515	0.127	-0.163	0.651
VO2max	-0.018	0.96	-0.139	0.7	-0.345	0.328	-0.309	0.384	-0.103	0.776	-0.357	0.31
Testosterone	-0.518	0.124	-0.396	0.256	-0.292	0.411	-0.359	0.307	-0.439	0.204	-0.189	0.6
FAI	-0.194	0.59	-0.084	0.816	-0.09	0.802	-0.129	0.72	-0.201	0.577	0.227	0.528
SHBG	-0.121	0.737	-0.273	0.444	-0.2	0.578	-0.255	0.476	-0.224	0.532	-0.504	0.136
LH	0.233	0.545	0.366	0.331	0.45	0.224	0.433	0.243	0.333	0.38	-0.183	0.636
FSH	0.05	0.897	0.041	0.914	0.267	0.486	0.242	0.529	0.192	0.619	-0.2	0.604
TC	0.548	0.1	0.5	0.141	0.408	0.241	0.347	0.325	0.292	0.411	-0.042	0.906
TG	0.634*	0.049	0.601	0.065	0.62	0.055	0.595	0.069	0.563	0.089	0.12	0.74
HDL	0.172	0.634	0.135	0.709	0.073	0.839	0.024	0.946	0.061	0.865	-0.455	0.185
LDL	0.42	0.225	0.439	0.204	0.298	0.401	0.28	0.432	0.182	0.612	0.609	0.0624
ALT	0.579	0.079	0.591	0.071	0.414	0.233	0.512	0.13	0.689*	0.027	0.176	0.625
HbA1c	0.242	0.529	0.192	0.619	0.276	0.471	0.317	0.404	0.326	0.391	0.075	0.847
TSH	-0.395	0.258	-0.291	0.413	-0.382	0.274	-0.316	0.373	-0.352	0.317	0.31	0.383
DHEAS	-0.263	0.528	-0.203	0.628	-0.359	0.382	-0.323	0.434	-0.275	0.509	0.526	0.179
Androsterone	-0.214	0.61	0	1	-0.214	0.61	-0.119	0.778	-0.119	0.778	0.547	0.16
Complement C1q	0.936**	0	0.942**	0	0.936**	0	0.924**	0	0.888**	0	0.182	0.614
Complement C3	1		0.964**	0	0.891**	0	0.903**	0	0.915**	0	0.248	0.488
Complement C3b/iC3b	-0.261	0.53	-0.261	0.53	-0.357	0.385	-0.333	0.419	-0.357	0.385	0.571	0.138
Complement C4	0.964**	0	1		0.927**	0	0.952**	0	0.939**	0	0.333	0.346
Complement factor-B	0.891**	0	0.927**	0	1		0.988**	0	0.927**	0	0.139	0.7
Complement factor-H	0.903**	0	0.952**	0	0.988**	0	1		0.964**	0	0.2	0.579
Properdin	0.915**	0	0.939**	0	0.927**	0	0.964**	0	1		0.139	0.7
complement C2	-0.187	0.603	-0.212	0.556	-0.054	0.881	-0.042	0.907	-0.139	0.7	0.139	0.7
Complement C4b	0.248	0.488	0.333	0.346	0.139	0.7	0.2	0.579	0.139	0.7	1	
Complement C5	0.078	0.828	0.127	0.726	-0.09	0.802	-0.078	0.828	-0.066	0.854	0.321	0.365
Complement C5a	-0.404	0.319	-0.333	0.419	-0.095	0.822	-0.095	0.822	-0.19	0.651	0.166	0.693
complement factor-D	-0.127	0.726	-0.054	0.881	-0.272	0.445	-0.236	0.51	-0.187	0.603	0.175	0.627
Manose-binding lectin	-0.345	0.328	-0.284	0.425	-0.406	0.244	-0.3697	0.293	-0.26	0.467	0.042	0.907
complement factor-1	0.2	0.579	0.248	0.488	0.042	0.907	0.054	0.881	0.03	0.933	0.43	0.214

r is Spearman rank correlation coefficient. *P<0.05, **P<0.001 is considered to be statistically significant. BMI, Body Mass Index; WHR, Waist Hip ratio; SBP, Systolic blood pressure; DBP, Diastolic blood pressure; NEFA, non, esterified free fatty acids; CHO, cholesterol; M value,insulin sensitivity; VO2max, maximum amount of oxygen utilized during exercise; FAI, free androgen index; SHBG, Sex hormone binding globulin; LH, luteinizing hormone; FSH, follicle stimulating hormone; TC, Total cholesterol; LDL, Low density lipoprotein cholesterol and HDL, High density lipoprotein cholesterol; TG, triglyceride; ALT, alanine transferase; FPG, fasting plasma glucose; HbA1c glycated hemoglobin; TSH, thyroid stimulating hormone; DHEAS, Dehydroepiandrosterone.

**Table 3B:** Spearman rank correlation analysis of complement related proteins with anthropometric and hormonal variables before exercise in PCOS subjects.

	Complement C-3		Complement C4		Factor-B		Factor-H		Properdin		Complement C4b	
	r	p	r	p	r	p	r	p	r	p	r	p
Age	0.115	0.751	0.163	0.651	-0.2	0.579	0.139	0.7	0.066	0.854	-0.09	0.802
Height	-0.165	0.647	-0.496	0.143	-0.288	0.419	-0.447	0.194	-0.263	0.461	-0.276	0.44
Weight	0.296	0.404	0.175	0.627	0.018	0.96	0.03	0.933	0.163	0.651	0.139	0.7
BMI	0.333	0.346	0.442	0.2	0.054	0.881	0.272	0.445	0.345	0.328	0.345	0.328
Waist	0.321	0.365	0.284	0.425	-0.018	0.96	0.127	0.726	0.272	0.445	0.054	0.881
Hip	0.447	0.194	0.325	0.359	0.128	0.722	0.263	0.461	0.361	0.304	0.165	0.647
WHR	0.163	0.651	0.187	0.603	-0.163	0.651	0.054	0.881	0.127	0.726	-0.03	0.933
SBP	0.648*	0.042	0.758*	0.011	0.624	0.053	0.782**	0.007	0.648*	0.042	0.345	0.328
DBP	0.146	0.686	0.408	0.241	0.317	0.372	0.219	0.542	-0.036	0.92	0.152	0.674
PG	-0.146	0.685	0.183	0.611	0.128	0.723	0.128	0.723	-0.146	0.685	0	1
Insulin	0.153	0.695	0.237	0.539	0.034	0.931	0.203	0.6	0.271	0.48	0.356	0.347
NEFA	-0.333	0.346	0.03	0.933	0.163	0.651	-0.006	0.986	-0.357	0.31	0.406	0.244
M-Value	0.115	0.751	-0.078	0.828	-0.042	0.907	0.151	0.676	0.454	0.186	-0.163	0.651
VO2max	-0.321	0.365	-0.454	0.186	-0.078	0.828	-0.296	0.404	-0.466	0.173	-0.151	0.676
Testosterone	0.393	0.259	-0.127	0.726	-0.078	0.828	-0.139	0.7	0.151	0.676	-0.393	0.259
FAI	-0.098	0.786	-0.196	0.585	0.036	0.919	-0.196	0.585	-0.135	0.709	0.147	0.683
SHBG	0.487	0.152	0.298	0.401	0.207	0.565	0.25	0.486	0.164	0.649	-0.079	0.827
LH	0.296	0.404	0.054	0.881	-0.2	0.579	0.006	0.986	-0.103	0.776	-0.2	0.579
FSH	0.369	0.293	0.466	0.173	0.127	0.726	0.333	0.346	0.078	0.828	0.224	0.533
TC	-0.316	0.373	-0.827**	0.003	-0.498	0.142	-0.699*	0.024	-0.577	0.08	-0.607	0.062
TG	0.072	0.841	-0.352	0.317	-0.012	0.973	-0.121	0.737	-0.121	0.737	-0.291	0.413
HDL	-0.361	0.304	-0.558	0.093	-0.19	0.598	-0.3	0.398	-0.263	0.461	-0.46	0.18
LDL	0.018	0.96	-0.164	0.65	0.097	0.789	-0.279	0.433	-0.34	0.335	-0.279	0.433
ALT	0.758	0.181	-0.566	0.111	-0.466	0.205	-0.416	0.264	-0.1	0.797	-0.717*	0.029
HbA1c	-0.149	0.679	-0.386	0.269	-0.249	0.487	-0.186	0.604	-0.261	0.465	0.099	0.784
TSH	-0.291	0.413	-0.583	0.076	-0.297	0.403	-0.541	0.106	-0.237	0.509	-0.553	0.097
DHEAS	0.2	0.579	0.139	0.7	0.115	0.751	0.187	0.603	0.551	0.098	-0.127	0.726
Androsterone	0.03	0.933	-0.26	0.467	-0.406	0.244	-0.224	0.533	0.175	0.627	-0.575	0.081
Complement- C1q	0.830**	0.002	0.855**	0.001	0.697*	0.025	0.915**	0	0.927**	0	0.309	0.384
Complement C-3	1		0.685*	0.028	0.527	0.117	0.782**	0.007	0.842**	0.002	0.139	0.7
Complement C3b/iC3b	0.333	0.346	0.43	0.214	0.369	0.293	0.49	0.149	0.478	0.161	0.515	0.127
Complement C4	0.685*	0.028	1		0.818**	0.003	0.927**	0	0.745*	0.013	0.648*	0.042
Complement factor-B	0.527	0.117	0.818**	0.003	1		0.830**	0.002	0.551	0.098	0.636*	0.047
Complement factor-H	0.782**	0.007	0.927**	0	0.830**	0.002	1		0.842**	0.002	0.587	0.073
Properdin	0.842**	0.002	0.745*	0.013	0.551	0.098	0.842**	0.002	1		0.236	0.51
complement C2	-0.624	0.053	-0.212	0.556	0.03	0.933	-0.321	0.365	-0.49	0.149	0.139	0.7
Complement C4b	0.139	0.7	0.648*	0.042	0.636*	0.047	0.587	0.073	0.236	0.51	1	
Complement C5	0.09	0.802	0.151	0.676	0.43	0.214	0.212	0.556	-0.163	0.651	0.296	0.404
Complement C5a	-0.212	0.555	-0.03	0.933	-0.2	0.578	-0.103	0.776	-0.03	0.933	0.322	0.363
complement factor-D	-0.321	0.365	-0.272	0.445	-0.01	0.96	-0.296	0.404	-0.345	0.328	-0.151	0.676
Manose-binding lectin	-0.078	0.828	-0.2	0.579	-0.43	0.214	-0.151	0.676	0.078	0.828	-0.406	0.244
complement factor-1	0.369	0.293	0.442	0.2	0.770**	0.009	0.515	0.127	0.224	0.533	0.393	0.259

r is Spearman rank correlation coefficient. *P<0.05, **P<0.001 is considered to be statistically significant. BMI, Body Mass Index; WHR, Waist Hip ratio; SBP, Systolic blood pressure; DBP, Diastolic blood pressure; NEFA, non, esterified free fatty acids; CHO, cholesterol; M value,insulin sensitivity; VO2max, maximum amount of oxygen utilized during exercise; FAI, free androgen index; SHBG, Sex hormone binding globulin; LH, luteinizing hormone; FSH, follicle stimulating hormone; TC, Total cholesterol; LDL, Low density lipoprotein cholesterol and HDL, High density lipoprotein cholesterol; TG, triglyceride; ALT, alanine transferase; FPG, fasting plasma glucose; HbA1c glycated hemoglobin; TSH, thyroid stimulating hormone; DHEAS, Dehydroepiandrosterone.

### Correlation Analysis of Complement-Related Proteins With Its Family Members Before Exercise

In control subjects, complement C3 was positively associated with complement C1q (*P* = 0.001), C4 (*P* < 0.0001), factor B (*P* < 0.0001), factor H (*P* < 0.0001), and properdin (*P* = 0.0001). Complement C4 was positively associated with C1q (*P* < 0.0001), C3 (*P* < 0.0001), factor B (*P* < 0.0001), factor H (*P* < 0.0001), and properdin (*P* < 0.0001). Factor B was positively associated with C1q (*P* < 0.0001), C3 (*P* < 0.0001), C4 (*P* < 0.0001), factor H (*P* < 0.0001), and properdin (*P* < 0.0001). Similarly, factor H was positively associated with C1q (*P* < 0.0001), C3 (*P* < 0.0001), C4 (*P* < 0.0001), factor B (*P* < 0.0001), and properdin (*P* < 0.0001). Properdin was associated positively with C1q (*P* < 0.0001), C3 (*P* = 0.001), C4 (*P* < 0.0001), factor B (*P* < 0.0001), and factor H (*P* < 0.0001). Complement C4b did not show any significant associations with clinical or biochemical parameters measured in control subjects (**Table 3A**).

In PCOS subjects, complement C3 was positively associated with complement C1q (*P* = 0.002), C4 (*P* < 0.028), factor H (*P* < 0.007), and properdin (*P* = 0.002). Complement C4 was positively associated with C1q (*P* = 0.001), C3 (*P* = 0.028), factor B (*P* = 0.003), factor H (*P* < 0.0001), properdin (*P* = 0.013), and C4b (*P* = 0.042). Factor B was positively associated with C1q (*P* = 0.025), C4 (*P* = 0.003), factor H (*P* = <0.002), C4b (*P* = 0.047), and complement factor 1 (*P* = 0.009). Similarly, factor H was positively associated with C1q (*P* < 0.0001), C3 (*P* = 0.007), C4 (*P* < 0.0001), factor B (*P* = 0.002), and properdin (*P* = 0.002). Properdin was associated positively with C1q (*P* < 0.0001), C3 (*P* = 0.002), C4 (*P* = 0.013), and factor H (*P* = 0.002). Complement C4b showed a positive association with C4 (*P* = 0.042) and factor B (*P* = 0.047) in PCOS subjects (**Table 3B**).

### Correlation Analysis of Complement-Related Proteins With Covariates After Exercise

In the control group, complement C3 (*P* = 0.034), C4 (*P* = 0.010), factor B (*P* = 0.030), factor H (*P* = 0.012), and properdin (*P* = 0.012) showed positive association with waist circumference (**Table 3C**). In PCOS subjects, complement C3 negatively correlated with VO_2max_ (*P* = 0.026). C4 negatively correlated with VO_2max_ (*P* = 0.001) and TC (*P* = 0.030). Factor B (*P* = 0.039) and factor H (*P* = 0.007) also showed a negative correlation with VO_2max_. Complement properdin showed a negative correlation with VO_2max_ (*P* = 0.009) and a positive correlation with FSH (*P* = 0.035) in the PCOS group (**Table 3D**).

**Table 3C:** Spearman rank correlation analysis of complement related proteins with anthropometric and hormonal variables after exercise controls.

Control	Complement C3		Complement C4		Factor-B		Factor-H		Properdin		Complement C4b	
	r	p	r	p	r	p	r	p	r	p	r	p
Age	0.358	0.31	0.224	0.533	0.273	0.446	0.188	0.603	0.103	0.777	0.006	0.987
Height	-0.128	0.725	-0.073	0.841	-0.043	0.907	0.055	0.881	0.109	0.763	0.055	0.881
Weight	0.406	0.244	0.564	0.09	0.479	0.162	0.552	0.098	0.515	0.128	0.6	0.067
BMI	0.37	0.293	0.527	0.117	0.479	0.162	0.527	0.117	0.467	0.174	0.588	0.074
Waist	0.669*	0.034	0.767**	0.01	0.681*	0.03	0.755*	0.012	0.755*	0.012	0.62	0.056
Hip	0.467	0.174	0.624	0.054	0.552	0.098	0.612	0.06	0.552	0.098	0.442	0.2
WHR	0.455	0.187	0.37	0.293	0.212	0.556	0.273	0.446	0.382	0.276	0.539	0.108
SBP	0.309	0.385	0.333	0.347	0.503	0.138	0.491	0.15	0.37	0.293	0.103	0.777
DBP	0.377	0.283	0.255	0.476	0.255	0.476	0.225	0.532	0.158	0.663	0.261	0.466
PG	-0.086	0.872	-0.029	0.957	0.086	0.872	0.086	0.872	-0.143	0.787	0.086	0.872
Insulin	0.395	0.439	0.516	0.295	0.698	0.123	0.698	0.123	0.577	0.231	-0.03	0.954
NEFA	-0.042	0.907	-0.042	0.907	0.042	0.907	-0.067	0.855	-0.152	0.676	-0.515	0.128
M-Value	-0.321	0.365	-0.333	0.347	-0.418	0.229	-0.442	0.2	-0.382	0.276	-0.2	0.58
VO2max	0.03	0.934	-0.042	0.907	-0.285	0.425	-0.236	0.511	-0.018	0.96	-0.042	0.907
Testosterone	-0.366	0.298	-0.28	0.432	-0.299	0.402	-0.341	0.334	-0.348	0.325	-0.47	0.171
FAI	-0.247	0.491	-0.095	0.794	-0.203	0.574	-0.158	0.662	-0.139	0.701	0.158	0.662
SHBG	-0.122	0.738	-0.237	0.51	-0.188	0.602	-0.249	0.487	-0.231	0.521	-0.529	0.116
LH	0.15	0.7	0.217	0.576	-0.083	0.831	-0.05	0.898	0.017	0.966	0.267	0.488
FSH	-0.233	0.546	-0.033	0.932	-0.217	0.576	-0.167	0.668	-0.2	0.606	0.45	0.224
TC	0.48	0.16	0.468	0.172	0.426	0.22	0.347	0.327	0.219	0.544	0.158	0.663
TG	0.024	0.947	0.055	0.881	0.237	0.51	0.152	0.675	0.03	0.934	-0.024	0.947
HDL	0.019	0.959	0.025	0.946	-0.043	0.906	-0.093	0.799	-0.08	0.826	-0.383	0.275
LDL	0.354	0.316	0.323	0.362	0.207	0.565	0.159	0.662	0.079	0.828	0.171	0.637
ALT	-0.17	0.638	-0.128	0.725	-0.231	0.521	-0.207	0.567	-0.14	0.7	-0.207	0.567
HbA1c	0.38	0.313	0.329	0.387	0.295	0.44	0.329	0.387	0.321	0.4	0.447	0.227
TSH	-0.685	0.09	-0.45	0.31	-0.577	0.175	-0.577	0.175	-0.667	0.102	0.468	0.289
DHEAS	-0.142	0.715	0.008	0.983	0.134	0.731	0.176	0.651	0.117	0.764	0.109	0.781
Androsterone	-0.393	0.295	-0.167	0.667	-0.167	0.667	-0.151	0.699	-0.209	0.589	-0.142	0.715
Complement C1q	0.936**	0	0.942**	0	.936**	0	0.924**	0	0.888**	0.001	0.182	0.614
Complement C3	1	.	0.964**	0	.891**	0.001	0.903**	0	0.915**	0	0.248	0.489
Complement C3b/iC3b	-0.262	0.531	-0.262	0.531	-0.357	0.385	-0.333	0.42	-0.357	0.385	0.571	0.139
Complement C4	0.964**	0	1	.	0.927**	0	0.952**	0	0.939**	0	0.333	0.347
Factor-B	0.891**	0.001	0.927**	0	1	.	0.988**	0	0.927**	0	0.139	0.701
Factor-H	0.903**	0	0.952**	0	0.988**	0	1	.	0.964**	0	0.2	0.58
Properdin	0.915**	0	0.939**	0	0.927**	0	0.964**	0	1	.	0.139	0.701
Complement C2	-0.188	0.603	-0.212	0.556	-0.055	0.881	-0.042	0.907	-0.139	0.701	0.139	0.701
Complement C4b	0.248	0.489	0.333	0.347	0.139	0.701	0.2	0.58	0.139	0.701	1	.
Complement C5	0.079	0.829	0.127	0.726	-0.091	0.803	-0.079	0.829	-0.067	0.855	0.321	0.365
Complement C5a	-0.405	0.32	-0.333	0.42	-0.095	0.823	-0.095	0.823	-0.19	0.651	0.167	0.693
Complement Factor-D	-0.127	0.726	-0.055	0.881	-0.273	0.446	-0.236	0.511	-0.188	0.603	0.176	0.627
Manose-binding lectin	-0.345	0.328	-0.285	0.425	-0.406	0.244	-0.37	0.293	-0.261	0.467	0.042	0.907
Complement factor-1	0.2	0.58	0.248	0.489	0.042	0.907	0.055	0.881	0.03	0.934	0.43	0.214

r is Spearman rank correlation coefficient. *P<0.05; **P<0.001 is considered to be statistically significant. BMI, Body Mass Index; WHR, Waist Hip ratio; SBP, Systolic blood pressure; DBP, Diastolic blood pressure; NEFA, non, esterified free fatty acids; CHO, cholesterol; M value,insulin sensitivity; VO2max, maximum amount of oxygen utilized during exercise; FAI, free androgen index; SHBG, Sex hormone binding globulin; LH, luteinizing hormone; FSH, follicle stimulating hormone; TC, Total cholesterol; LDL, Low density lipoprotein cholesterol and HDL, High density lipoprotein cholesterol; TG, triglyceride; ALT, alanine transferase; FPG, fasting plasma glucose; HbA1c glycated hemoglobin; TSH, thyroid stimulating hormone; DHEAS, Dehydroepiandrosterone.

**Table 3D:** Spearman rank correlation analysis of complement related proteins with anthropometric and hormonal variables after exercise PCOS subjects.

PCOS	Complement C3		Complement C4		Factor-B		Factor-H		Properdin		Complement C4b	
	r	p	r	p	r	p	r	p	r	p	r	p
Age	0.115	0.751	0.164	0.651	-0.2	0.58	0.139	0.701	0.067	0.855	-0.091	0.803
Height	-0.166	0.647	-0.497	0.144	-0.288	0.419	-0.448	0.194	-0.264	0.461	-0.276	0.44
Weight	0.418	0.229	0.333	0.347	0.127	0.726	0.212	0.556	0.333	0.347	0.273	0.446
BMI	0.382	0.276	0.455	0.187	0.127	0.726	0.297	0.405	0.467	0.174	0.261	0.467
Waist	0.494	0.147	0.402	0.249	0.195	0.589	0.28	0.432	0.421	0.226	0.073	0.841
Hip	0.498	0.143	0.462	0.179	0.267	0.455	0.371	0.291	0.432	0.213	0.231	0.521
WHR	0.2	0.58	0.188	0.603	-0.018	0.96	0.018	0.96	0.224	0.533	-0.103	0.777
SBP	0.687*	0.028	0.657*	0.039	0.657*	0.039	0.571	0.084	0.626	0.053	0.122	0.738
DBP	0.573	0.083	0.372	0.29	0.274	0.443	0.451	0.191	0.463	0.177	0.024	0.947
PG	0.096	0.82	-0.12	0.776	-0.193	0.647	0.048	0.91	0.229	0.586	-0.181	0.668
Insulin	0.071	0.867	0.31	0.456	0.071	0.867	0.286	0.493	0.333	0.42	0.381	0.352
NEFA	0.43	0.214	0.576	0.082	0.442	0.2	0.467	0.174	0.236	0.511	0.491	0.15
M-Value	-0.37	0.293	-0.358	0.31	-0.188	0.603	-0.309	0.385	-0.152	0.676	-0.442	0.2
VO2max	-0.693*	0.026	-0.875**	0.001	-0.693*	0.026	-0.784**	0.007	-0.772**	0.009	-0.559	0.093
Testosterone	0.272	0.448	-0.29	0.416	-0.154	0.67	-0.179	0.621	0.247	0.492	-0.525	0.119
FAI	0.08	0.827	0.226	0.53	-0.012	0.973	0.073	0.84	0.245	0.496	0.098	0.788
SHBG	0.14	0.699	-0.232	0.519	0.006	0.987	-0.104	0.776	-0.14	0.699	-0.329	0.353
LH	0.033	0.932	-0.2	0.606	-0.567	0.112	-0.183	0.637	0.217	0.576	-0.417	0.265
FSH	0.385	0.306	0.519	0.152	0.151	0.699	0.452	0.222	0.703*	0.035	0.318	0.404
TC	-0.141	0.697	-0.681*	0.03	-0.264	0.461	-0.448	0.194	-0.301	0.399	-0.62	0.056
TG	0.127	0.726	-0.285	0.425	0.127	0.726	-0.018	0.96	-0.018	0.96	-0.273	0.446
HDL	-0.265	0.46	-0.505	0.137	-0.302	0.397	-0.283	0.428	-0.166	0.646	-0.425	0.221
LDL	-0.195	0.59	-0.535	0.111	-0.261	0.466	-0.498	0.143	-0.28	0.434	-0.432	0.213
ALT	0.435	0.242	0	1	-0.1	0.797	-0.1	0.797	-0.151	0.699	-0.285	0.458
HbA1c	-0.144	0.758	-0.505	0.248	-0.126	0.788	-0.09	0.848	-0.162	0.728	-0.126	0.788
TSH	-0.41	0.273	-0.159	0.683	-0.184	0.635	-0.351	0.354	-0.351	0.354	0.1	0.797
DHEAS	-0.252	0.548	0.395	0.333	0.132	0.756	0.192	0.649	0.192	0.649	0.359	0.382
Androsterone	0.107	0.819	0.464	0.294	0.286	0.535	0.607	0.148	0.75	0.052	0.286	0.535
Complement- C1q	0.830**	0.003	0.855**	0.002	0.697*	0.025	0.915**	0	0.927**	0	0.309	0.385
Complement C-3	1	.	0.685*	0.029	0.527	0.117	0.782**	0.008	0.842**	0.002	0.139	0.701
Complement C3b/iC3b	0.333	0.347	0.43	0.214	0.37	0.293	0.491	0.15	0.479	0.162	0.515	0.128
Complement C4	0.685*	0.029	1	.	0.818**	0.004	0.927**	0	0.745*	0.013	0.648*	0.043
Factor-B	0.527	0.117	0.818**	0.004	1	.	0.830**	0.003	0.552	0.098	0.636*	0.048
Factor-H	0.782**	0.008	0.927**	0	0.830**	0.003	1	.	0.842**	0.002	0.588	0.074
Properdin	0.842**	0.002	0.745*	0.013	0.552	0.098	0.842**	0.002	1	.	0.236	0.511
complement C2	-0.624	0.054	-0.212	0.556	0.03	0.934	-0.321	0.365	-0.491	0.15	0.139	0.701
Complement C4b	0.139	0.701	0.648*	0.043	0.636*	0.048	0.588	0.074	0.236	0.511	1	.
Complement C5	0.091	0.803	0.152	0.676	0.43	0.214	0.212	0.556	-0.164	0.651	0.297	0.405
Complement C5a	-0.213	0.555	-0.03	0.934	-0.201	0.578	-0.103	0.776	-0.03	0.934	0.322	0.364
Complement Factor D	-0.321	0.365	-0.273	0.446	-0.018	0.96	-0.297	0.405	-0.345	0.328	-0.152	0.676
Manose-binding lectin	-0.079	0.829	-0.2	0.58	-0.43	0.214	-0.152	0.676	0.079	0.829	-0.406	0.244
Complement factor-1	0.37	0.293	0.442	0.2	0.770**	0.009	0.515	0.128	0.224	0.533	0.394	0.26

r is Spearman rank correlation coefficient. *P<0.05, **P<0.001 is considered to be statistically significant. BMI, Body Mass Index; WHR, Waist Hip ratio; SBP, Systolic blood pressure; DBP, Diastolic blood pressure; NEFA, non, esterified free fatty acids; CHO, cholesterol; M value,insulin sensitivity; VO2max, maximum amount of oxygen utilized during exercise; FAI, free androgen index; SHBG, Sex hormone binding globulin; LH, luteinizing hormone; FSH, follicle stimulating hormone; TC, Total cholesterol; LDL, Low density lipoprotein cholesterol and HDL, High density lipoprotein cholesterol; TG, triglyceride; ALT, alanine transferase; FPG, fasting plasma glucose; HbA1c glycated hemoglobin; TSH, thyroid stimulating hormone; DHEAS, Dehydroepiandrosterone.

### Correlation Analysis of Complement-Related Proteins With Its Family Members After Exercise Intervention

In control subjects, complement C3 was positively associated with complement C1q (*P* < 0.0001), C4 (*P* < 0.0001), factor B (*P* = 0.001), factor H (*P* < 0.0001), and properdin (*P* < 0.0001). Complement C4 was positively associated with C1q (*P* < 0.0001), C3 (*P* < 0.0001), factor B (*P* < 0.0001), factor H (*P* < 0.0001), and properdin (*P* < 0.0001). Factor B was positively associated with C1q (*P* < 0.0001), C3 (*P* = 0.001), C4 (*P* < 0.0001), factor H (*P* < 0.0001), and properdin (*P* < 0.0001). Similarly, factor H was positively associated with C1q (*P* < 0.0001), C3 (*P* < 0.0001), C4 (*P* < 0.0001), factor B (*P* < 0.0001), and properdin (*P* < 0.0001). Properdin was associated positively with C1q (*P* = 0.001), C3 (*P* < 0.0001), C4 (*P* < 0.0001), factor B (*P* < 0.0001), and factor H (*P* < 0.0001). Complement C4b did not show any association with clinical or biochemical variables in the control group of subjects (**Table 3C**). In PCOS subjects, C3 was positively associated with complement C1q (*P* = 0.003), C4 (*P* = 0.029), factor H (*P* = 0.008), and properdin (*P* = 0.002). Complement C4 was positively associated with C1q (*P* = 0.002), C3 (*P* = 0.0293), factor B (*P* = 0.004), factor H (*P* < 0.0001), properdin (*P* = 0.013), and C4b (*P* = 0.043). Factor B was positively associated with C1q (*P* = 0.025), C4 (*P* = 0.004), factor H (*P* = 0.003), C4b (*P* = 0.048), and complement factor 1 (*P* = 0.009). Similarly, factor H was positively associated with C1q (*P* < 0.0001), C3 (*P* = 0.008), C4 (*P* < 0.0001), factor B (*P* = 0.003), and properdin (*P* = 0.002). Properdin was associated positively with C1q (*P* < 0.0001), C3 (*P* = 0002), C4 (*P* = 0.013), and factor H (*P* = 0.002). Complement C4b was associated positively with C4 (*P* = 0.043) and factor B (*P* = 0.048, **Table 3D**).

## Discussion

The complement system plays important roles in immunity and inflammation (8). Obesity and PCOS are characterized by dysregulation of several components of the complement system (9). Aerobic exercises were shown to induce the activation of the alternative pathway of the complement system (19). Our study shows that moderate aerobic exercise improves insulin sensitivity and cardiometabolic fitness in both the control and the PCOS subjects; however, it predominantly reduced the components of the complement system in the control subjects, but not in the subjects with PCOS.

At baseline, we found significantly elevated complement C4b in PCOS subjects compared with controls. However, no significant differences were observed between controls and PCOS subjects for complement factor I, C1q, C2, C3, C3b/iC3b, C4, C5, C5a, complement factor D, mannose-binding lectin, factor B, factor H, and properdin. Our findings are consistent with some (17, 18) but not all (14, 16) previously reported studies, where no differences in C3 level in women with PCOS compared with controls were found. These differences may be due to the relationship of the complement proteins to insulin resistance and obesity (9) as PCOS women were weight- and age-matched to the controls with no difference in the baseline insulin resistance, and therefore, those complement changes due to these parameters would have been taken into account.

**Figure 1:**
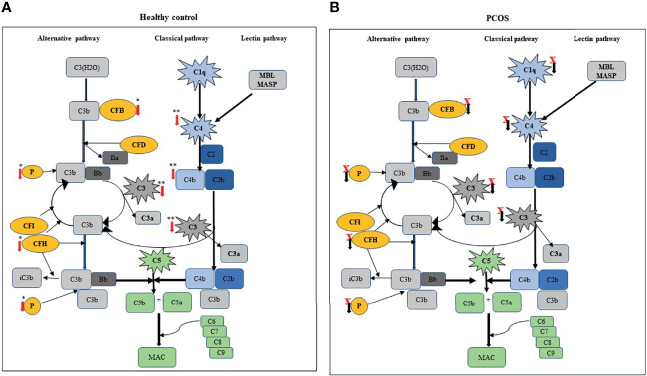
Comparison of exercise-induced changes in complement-related proteins between control and PCOS subjects: **(A)** schematic represents the level of secreted complement components, such as C1q, C3, C3b/iC3b, C4, and FB; FH, properdin (P), and C4b were significantly decreased after exercise in a healthy subject. **(B)** In PCOS subjects, circulating component-related proteins were not significantly altered following exercise. Red (↓) arrow indicates the complement-related proteins that are reduced following exercise. In the PCOS group, black (↓) arrow with a red cross indicates no significant difference following exercise.

In this study, moderate aerobic exercise showed a significant reduction of complement-related protein family members both within-group (C1q, C3, and factor H) and between-group (C3, C4, factor B, factor H, properdin, and C4b) analyses. However, these changes appear to be predominantly restricted to the control group of subjects. In the PCOS group, exercise did not change the levels of the secreted complement components C1q, C3, and factor H, and therefore, it appears that complement pathways were not activated in PCOS subjects following moderate exercise. This may be due to dysregulation of the complement system in women with PCOS (**Figure 1B**). In comparison, members of complement proteins C1q, C3, and factor H were significantly downregulated following exercise in controls (**Figure 1A**). The downregulation of these complement proteins following exercise may make individuals more susceptible to infections, particularly C3 (10). The significant decreases in C3 protein in the control group were similar to those reported in previous studies (32–34). The effect of the complement system on the immune system has a wide range of biological consequences, implying a wide range of relationships. It is thought that high-intensity physical exertion impairs the immune system of an organism (20, 35, 36) and that their long-term effects might lead to immunosuppression (20, 21). Regular, moderate-intensity physical exercise, on the other hand, promotes development and improves immunity (20, 35).

In our study, complement C3 and C4, as well as factor B, factor H, properdin, and C4b levels, were significantly different between groups after exercise. Assessment of the relationships between these complement-related proteins with anthropometric and hormonal characteristics of the subjects were in accord with the relationship of complement proteins with weight and BMI (9); we found a positive correlation for waist circumference in the control group of subjects. VO_2max_ is a factor that determines cardiometabolic status, and here VO_2max_ negatively correlated with complement C3 and C4b, as well as factor B, factor H, and properdin levels, suggesting that lower VO_2max_ (i.e., more unfit) was associated with higher complement levels at baseline. Similarly, another cardiometabolic-related factor SBP showed significant positive correlations following exercise, with complement factors C3, C4, and factor B in the PCOS group suggesting the link between complement factors and cardiovascular risk, which is usually higher in PCOS subjects. As expected, most of the family members of complement-related proteins regulate each other; therefore, significant positive correlations of complement-related proteins and its family members were seen both at baseline and following exercise.

The limitations of this study include the small number of participants from one single ethnicity (Caucasian) and did not account for metabolic differences and phenotypic differences that usually exists in PCOS subjects. Therefore, the findings may not be generalized to the wider population or to different ethnicities.

In conclusion, our data showed that exercise reduced several components of the complement system—C1q, C3, C4, factor B, factor H, and properdin—in control subjects, but not in PCOS women, although they were age- and weight-matched. The data suggest that the component system pathways remain dysregulated after moderate aerobic exercise in PCOS compared with control subjects, although insulin sensitivity after exercise was improved in both groups.

## Data Availability Statement

The original contributions presented in the study are included in the article/supplementary material. Further inquiries can be directed to the corresponding author.

## Ethics Statement

The studies involving human participants were reviewed and approved by the Yorkshire and the Humber Research Ethics Committee (reference number 10/H1313/44) and The Medical Research Center at Hamad Medical Corporation (reference number RP #17180/17). All study participants gave their written informed consent prior to participation in the study. The patients/participants provided their written informed consent to participate in this study.

## Author Contributions

MR, IB, JJ, and MB performed the measurements and contributed to the manuscript. IA and MR wrote the manuscript. SA, TS, and MM recruited the subjects and were involved in sample collection and data analysis. MA researched the data and contributed to the manuscript. MR, A-BA-S, and SA conceptualized the study, designed the experiments, supervised the progress, analyzed the data, and approved the final version of the article. All the authors reviewed and revised the manuscript.

## Conflict of Interest

The authors declare that the research was conducted in the absence of any commercial or financial relationships that could be construed as a potential conflict of interest.

The handling editor declared a past collaboration with several of the authors (MR, IB, SA, A-BA-S).

## Publisher’s Note

All claims expressed in this article are solely those of the authors and do not necessarily represent those of their affiliated organizations, or those of the publisher, the editors and the reviewers. Any product that may be evaluated in this article, or claim that may be made by its manufacturer, is not guaranteed or endorsed by the publisher.

## References

[1] Ehrmann DA. Polycystic Ovary Syndrome. N Engl J Med (2005) 352(12):1223–36. doi: 10.1056/NEJMra041536 15788499

[2] Jayasena CN Franks S. The Management of Patients With Polycystic Ovary Syndrome. Nat Rev Endocrinol (2014) 10(10):624. doi: 10.1038/nrendo.2014.102 25022814

[3] Stepto NK Cassar S Joham AE Hutchison SK Harrison CL Goldstein RF. Women With Polycystic Ovary Syndrome Have Intrinsic Insulin Resistance on Euglycaemic–Hyperinsulaemic Clamp. Hum Reprod (2013) 28(3):777–84. doi: 10.1093/humrep/des463 23315061

[4] Kyrou I Weickert MO Randeva HS. Diagnosis and Management of Polycystic Ovary Syndrome (PCOS). In: Ajjan R Orme S, editors. Endocrinology and Diabetes. London: Springer (2015) 99–113. doi: 10.1007/978-1-4471-2789-5_13

[5] Kyrou I Randeva HS Tsigos C Kaltsas G Weickert MO. Clinical Problems Caused by Obesity. Endotext (2018).

[6] Randeva HS Tan BK Weickert MO Lois K Nestler JE Sattar N. Cardiometabolic Aspects of the Polycystic Ovary Syndrome. Endocrine Rev (2012) 33(5):812–41. doi: 10.1210/er.2012-1003 PMC3461136 22829562

[7] Carroll MC. A Protective Role for Innate Immunity in Autoimmune Disease. Clin Immunol (2000) 95(1):S30–8. doi: 10.1006/clim.1999.4813 10729235

[8] Walport MJ. Complement. N Engl J Med (2001) 344(14):1058–66. doi: 10.1056/NEJM200104053441406 11287977

[9] Lewis RD Narayanaswamy AK Farewell D Rees DA. Complement Activation in Polycystic Ovary Syndrome Occurs in the Postprandial and Fasted State and Is Influenced by Obesity and Insulin Sensitivity. Clin Endocrinol (2021) 94(1):74–84. doi: 10.1111/cen.14322 PMC9623543 32865246

[10] Matsuyama W Nakagawa M Takashima H Muranaga F Sano Y Osame M. Molecular Analysis of Hereditary Deficiency of the Third Component of Complement (C3) in Two Sisters. Intern Med (2001) 40(12):1254–8. doi: 10.2169/internalmedicine.40.1254 11813855

[11] Sarma JV Ward PA. The Complement System. Cell Tissue Res (2011) 343(1):227–35. doi: 10.1007/s00441-010-1034-0 PMC3097465 20838815

[12] Prohászka Z Kirschfink M Frazer-Abel A. Complement Analysis in the Era of Targeted Therapeutics. Mol Immunol (2018) 102:84–8. doi: 10.1016/j.molimm.2018.06.001 29933889

[13] Gursoy Calan O Calan M Yesil Senses P Unal Kocabas G Ozden E Sari KR. Increased Adipsin Is Associated With Carotid Intima Media Thickness and Metabolic Disturbances in Polycystic Ovary Syndrome. Clin Endocrinol (2016) 85(6):910–7. doi: 10.1111/cen.13157 27434652

[14] Wu Y Zhang J Wen Y Wang H Zhang M Cianflone K. Increased Acylation-Stimulating Protein, C-Reactive Protein, and Lipid Levels in Young Women With Polycystic Ovary Syndrome. Fertil Steril (2009) 91(1):213–9. doi: 10.1016/j.fertnstert.2007.11.031 18206145

[15] Oktenli C Ozgurtas T Dede M Sanisoglu YS Yenen MC Yesilova Z. Metformin Decreases Circulating Acylation-Stimulating Protein Levels in Polycystic Ovary Syndrome. Gynecological Endocrinol (2007) 23(12):710–5. doi: 10.1080/09513590701666571 18075846

[16] Yang S Li Q Song Y Tian B Cheng Q Qing H. Serum Complement C3 has a Stronger Association With Insulin Resistance Than High-Sensitivity C-Reactive Protein in Women With Polycystic Ovary Syndrome. Fertil Steril (2011) 95(5):1749–53. doi: 10.1016/j.fertnstert.2011.01.136 21316661

[17] Dehdashtihaghighat S Mehdizadehkashi A Arbabi A Pishgahroudsari M Chaichian S. Assessment of C-Reactive Protein and C3 as Inflammatory Markers of Insulin Resistance in Women With Polycystic Ovary Syndrome: A Case-Control Study. J Reprod Infertil (2013) 14(4):197. 24551574 PMC3911815

[18] Snyder ML Shields KJ Korytkowski MT Sutton-Tyrrell K Talbott EO. Complement Protein C3 and Coronary Artery Calcium in Middle-Aged Women With Polycystic Ovary Syndrome and Controls. Gynecological Endocrinol (2014) 30(7):511–5. doi: 10.3109/09513590.2014.895985 PMC4065194 24592986

[19] Kostrzewa-Nowak D Kubaszewska J Nowakowska A Nowak R. Effect of Aerobic and Anaerobic Exercise on the Complement System of Proteins in Healthy Young Males. J Clin Med (2020) 9(8):2357. doi: 10.3390/jcm9082357 PMC7464301 32717972

[20] Njernqn D. Exercise Immunology: Practical Applications. J Sports Med (1997) 18(1):91–100. doi: 10.1055/s-2007-972705 9129268

[21] Peake JM Neubauer O Della Gatta PA Nosaka K. Muscle Damage and Inflammation During Recovery From Exercise. J Appl Physiol (2017) 122(3):559–70. doi: 10.1152/japplphysiol.00971.2016 28035017

[22] Peake JM Neubauer O Walsh NP Simpson RJ. Recovery of the Immune System After Exercise. J Appl Physiol (2017) 122(5):1077–87. doi: 10.1152/japplphysiol.00622.2016 27909225

[23] Pedersen BK Hoffman-Goetz L. Exercise and the Immune System: Regulation, Integration, and Adaptation. Physiol Rev (2000). doi: 10.1152/physrev.2000.80.3.1055 10893431

[24] Liu X Zeng Z Zhao L Xiao W Chen P. Changes in Inflammatory and Oxidative Stress Factors and the Protein Synthesis Pathway in Injured Skeletal Muscle After Contusion. Exp Ther Med (2018) 15(2):2196–202. doi: 10.3892/etm.2017.5625 PMC5776558 29434825

[25] Aye MM Kilpatrick ES Aburima A Wraith KS Magwenzi S Spurgeon B. Acute Hypertriglyceridemia Induces Platelet Hyperactivity That Is Not Attenuated by Insulin in Polycystic Ovary Syndrome. J Am Heart Assoc (2014) 3(1):e000706. doi: 10.1161/JAHA.113.000706 24584741 PMC3959686

[26] Foster C Florhaug JA Franklin J Gottschall L Hrovatin LA Parker S. A New Approach to Monitoring Exercise Training. J Strength Conditioning Res (2001) 15(1):109–15. 11708692

[27] Kirk RJ Madden LA Peart DJ Aye MM Atkin SL Vince RV. Circulating Endothelial Microparticles Reduce in Concentration Following an Exercise Programme in Women With Polycystic Ovary Syndrome. Front Endocrinol (2019) 10:200. doi: 10.3389/fendo.2019.00200 PMC6450458 30984117

[28] Ramanjaneya M Bensila M Bettahi I Jerobin J Samra TA Aye MM. Dynamic Changes in Circulating Endocrine FGF19 Subfamily and Fetuin-A in Response to Intralipid and Insulin Infusions in Healthy and PCOS Women. Front Endocrinol (2020) 11:568500. doi: 10.3389/fendo.2020.568500 PMC7554576 33101202

[29] Halama A Aye MM Dargham SR Kulinski M Suhre K Atkin SL. Metabolomics of Dynamic Changes in Insulin Resistance Before and After Exercise in PCOS. Front Endocrinol (2019) 10:116. doi: 10.3389/fendo.2019.00116 PMC6400834 30873121

[30] Ramanjaneya M Butler AE Alkasem M Bashir M Jerobin J Godwin A. Association of Complement-Related Proteins in Subjects With and Without Second Trimester Gestational Diabetes. Front Endocrinol (2021) 12:641361. doi: 10.3389/fendo.2021.641361 PMC8043150 33859618

[31] Ramanjaneya M Jerobin J Bettahi I Bensila M Aye M Siveen KS. Lipids and Insulin Regulate Mitochondrial-Derived Peptide (MOTS-C) in PCOS and Healthy Subjects. Clin Endocrinol (Oxf) (2019) 91: (2):278–87. doi: 10.1111/cen.14007 31066084

[32] Karacabey K Saygin O Ozmerdivenli R Zorba E Godekmerdan A Bulut V. The Effects of Exercise on the Immune System and Stress Hormones in Sportswomen. Neuroendocrinol Lett (2005) 26(4):361–6. 16136008

[33] Mashiko T Umeda T Nakaji S Sugawara K. Position Related Analysis of the Appearance of and Relationship Between Post-Match Physical and Mental Fatigue in University Rugby Football Players. Br J Sports Med (2004) 38(5):617–21. doi: 10.1136/bjsm.2003.007690 PMC1724951 15388551

[34] Karacabey K Peker I Saygın Ö Cıloglu F Ozmerdivenli R Bulut V. Effects of Acute Aerobic and Anaerobic Exercise on Humoral Immune Factors in Elite Athletes. Biotechnol Biotechnol Equip (2005) 19(1):175–80. doi: 10.1080/13102818.2005.10817177

[35] Simpson RJ Kunz H Agha N Graff R. Exercise and the Regulation of Immune Functions. Prog Mol Biol Trans Sci (2015) 135:355–80. doi: 10.1016/bs.pmbts.2015.08.001 26477922

[36] Pedersen B Rohde T Zacho M. Immunity in Athletes. J Sports Med Phys fitness (1996) 36(4):236–45. 9062046

